# Anti-Opium Pill

**Published:** 1874-01

**Authors:** 


Anti-Opium Pill.—The following formula for an anti-opium pill has been employed for several years in the English hospitals at Pekin, and its efficacy proven in numerous instances:

| | |
|---|---|
| ℞.—Henbane, . . . . . | gr. one-quarter; |
| Gentian, . . . . . | gr. one-half; |
| Quinine, . . . . . | gr. one-quarter; |
| Ginger, . . . . . | gr. one-half; |
| Camphor, . . . . . | gr. one-half; |
| Cayenne, . . . . . | gr. one-half; |
| Cinnamon, . . . . . | gr. one-half. |

M. Three pills a day.—*London Druggist.*

Extraordinary Precocity.—Dr. A. R. Kilpatrick, of Navasota, reports (*Med. and Surg. Reporter*) that a negro girl in that county has given birth to a fully developed female child, at the early age of about *eleven and one-half years.*

